# The Main Issue

**Published:** 1915-11

**Authors:** 


### The Main Issue.

The main issue in medicine is medical science and practice, not medical politics, nor glory, nor even personal financial success. No fine point in medical etiquette or ethics—especially what is sometimes called ethics—is so important as the study of disease. Let each man ask himself which of these he is really most interested in. One of the best signs of the times is that, generally speaking, the largest attendance at medical meetings coincides with the best scientific and practical programs—with due regard for the attraction of outside talent—and no longer marks the anticipation of a fight for office or on some point of the by-laws.

